# Accidental ingestion of concentrated white vinegar in Hatay children in Turkey

**DOI:** 10.2478/aiht-2023-74-3792

**Published:** 2023-12-29

**Authors:** Ahmet Atıcı, Lina Miçooğulları, Bahar Uğur, Mehmet Emin Çelikkaya, Bülent Akçora

**Affiliations:** Mustafa Kemal University School of Medicine, Department of Paediatric Surgery, Hatay, Turkey

**Keywords:** acetic acid, bougie dilation, follow-up, oesophagus, postoperative complications, swallowing, bužija, dilatacija jednjaka, postoperativne komplikacije, praćenje, zaštitni čepovi

## Abstract

White vinegar which contains high concentrations (~85 %) of acetic acid is a staple ingredient used in food preparation in many Mediterranean cuisines but in small amounts. Being corrosive, it can cause ulcerative injury to the oropharynx and oesophagus and upset the stomach with resulting nausea and vomiting. This study presents 11 cases of paediatric patients (five boys and six girls, aged between 11 and 89 months) with oesophageal strictures who drank white vinegar by accident. They all received endoscopic oesophageal dilation (with a bougie) ranging from one to 28 per patient, depending on the severity of the injury. Follow-up showed uneventful healing in eight patients, who at the time of the telephone call were able to swallow solids and liquids normally. Two patients who could not be reached by telephone were found healthy by consulting the national database (e-Nabız). Unfortunately, one patient, who was discharged without any symptoms after the first dilation, suffered massive gastrointestinal bleeding 24 hours after the dilation and died. The loss of this patient shows that ingesting white vinegar can be very dangerous in children, especially if parents delay seeking medical help. We believe that controlling the production and sales of highly concentrated white vinegar and selling it in child-proof containers can help to prevent accidental ingestions by children and tragic outcomes such as the one reported here.

## Abstract (sl)

Bijeli ocat, koji sadrži visoke koncentracije (~85 %) octene kiseline, često se koristi u pripremi hrane u mediteranskoj kuhinji, no u malim količinama. U suprotnome može prouzročiti ulceroznu ozljedu usnog dijela ždrijela i jednjaka te izazvati želučane tegobe s povremenim mučninama i povraćanjem. Naš prikaz obuhvaća ukupno jedanaestero djece primljene na odjel od siječnja 2014. do prosinca 2022. zbog slučajnoga gutanja bijeloga octa (petero dječaka i šest djevojčica u dobnom rasponu od 11 do 89 mjeseci) koja su imala suženje jednjaka i prošla postupak dilatacije (pomoću bužije) od jednog do 28 puta. Ozdravljenje je nastavljeno bez ikakvih poteškoća u osmero od devetero bolesnika koji su u vrijeme telefonske provjere već mogli normalno gutati čvrstu hranu i tekućine. Za dvoje se bolesnika koji nisu bili dostupni telefonski ozdravljenje utvrdilo pretraživanjem državne baze podataka (e-Nabız). Jedan je bolesnik nakon prve dilatacije otpušten iz bolnice bez simptoma, no imao je masivno krvarenje u probavnom sustavu i preminuo 24 sata nakon dilatacije. U ostalih bolesnika nisu zabilježene daljnje postoperativne komplikacije. Naši podaci pokazuju da suženje jednjaka u djece izazvano gutanjem bijelog octa zahtijeva višekratni zahvat dilatacijom, ovisno o težini ozljede. No gubitak bolesnika koji je kasno započeo liječenje upozorava na činjenicu da gutanje bijelog octa može biti vrlo opasno u djece i da je potrebna hitna intervencija. Da bi se spriječili tragični ishodi poput ovoga, smatramo da je potrebno uvesti kontrolu proizvodnje i prodaje bijelog octa u visokim koncentracijama te na boce ugraditi zaštitne čepove.

Accidental ingestion of corrosive substances by children (most often aged 1–5 years) occurs at an annual rate of 5–518 per 100,000, usually involves household liquids, and the risk is often associated with low family socioeconomic status, poor parental supervision, and neurodevelopmental disorders (1, 2, 3, 4, 5, 6, 7, 8, 9, 10). The most common cause of mortality and morbidity in long-term follow-up of such patients are oesophageal strictures (3, 7, 10, 11, 12).

Aside from household liquids such as cleaners, there are corrosive substances used in food preparation (2, 5, 9, 13), most notably vinegar. Concentrated white vinegar is a colourless liquid often used in some Mediterranean cuisines, especially in less affluent countries. Considering its high concentration of acetic acid (~85 %) it is far more dangerous to ingest than wine vinegar (generally ~5 % acetic acid) and is seldom used in kitchens in developed countries (2, 9, 13, 14). In Turkey, it is often used to make homemade pickles, and being a clear, colourless liquid, children can easily mistake it for water if bottled or stored improperly.

During our daily practice, we have encountered many cases of children poisoning with concentrated white vinegar and therefore felt the need to raise awareness about this issue. The aim of this study is to present cases of paediatric patients with oesophageal strictures and report how the children were managed and followed-up after discharging them from the hospital.

The study was approved by the Ethics Committee of Mustafa Kemal University (decision No. 12 of 26 July 2023).

## DATA COLLECTION AND ANALYSIS

We reviewed all cases of children ingesting corrosive substances between January 2014 and December 2022 who were hospitalised at our Mustafa Kemal University Hospital Ppaediatric surgery clinic (Hatay, Turkey) and collected data regarding their age, gender, type of caustic substance, initial management, clinical findings, treatment, and complications. From these 182 records we selected 11 patients who developed oesophageal stricture/stenosis due to the ingestion of white vinegar and who received dilation treatment in that period. We also collected follow-up information from their parents/guardians concerning children's current health status. Patients with other comorbidities whose data could not be gathered and whose treatment was continued in other clinics were excluded from the study.

For descriptive statistics used in this study we relied on the SPSS statistical package version 18.0 (SPSS, Inc., Chicago, IL, USA). All numerical data are expressed as means (± standard deviation; SD).

## CASE SERIES

Patient histories and clinical examination confirmed that the ingested substance was white vinegar in all cases. In most of them the children mistook white vinegar in a bottle or a glass in the kitchen for water and swallowed one to two sips before they realised the mistake.

None of the patients underwent endoscopy in the early stage (24–48 h) of treatment, but all took routine laboratory tests, which proved normal. No systemic effects were observed in any of the patients. In those who had symptoms such as hypersalivation (which was the most common), feeding was replaced by intravenous fluid therapy, which also included antibiotics and proton pump inhibitors (PPI) for 48–72 h. None received steroid treatment. Posterior-anterior chest radiographs showed no pneumoperitoneum or pneumomediastinum, nor did any of the patients need gastrostomy.

Patients whose oropharyngeal oedema regressed, who could swallow their saliva, and who could swallow liquid food were started on a soft diet for 24–48 h and later discharged with the intention to run a follow-up passage radiography (oesophagus-stomach-duodenum contrast X-rays) in three weeks. In ten patients passage radiography revealed stenosis in one place and in one in two places. None of the patients had a gastric outlet obstruction or gastric injury. All underwent antegrade dilation with a guide wire under general anaesthesia. All patients who had no complications such as fistulas, perforations, or bleeding were discharged on the same day. At first dilations were performed once a week or every two weeks, depending on the patient's clinic. As the severity of the initial stenosis eased and clinical response was achieved, the intervals gradually increased to 3–4 weeks. During dilation, all patients manifested a circularly narrowed oesophageal stenosis, but its anatomy was normal in the proximal part of the stenosis. If minimal bleeding was encountered during dilation, the procedure was stopped. After reaching the dilator size suitable for the patient age, dilation intervals gradually increased to 6–8 weeks. The number of interventions depended on the clinical course. Altogether, the patients underwent between one and 28 dilations (1–30) months in average.

In the follow-up stage, which lasted between six and 144 months, parents/relatives of nine patients were contacted by phone and for two the information was found on the national database (e-Nabız). Ten patients healed uneventfully, had no postoperative complications and recovered completely, but one died. The child was brought to our clinic as late as 20 days after she drank the vinegar and could no longer swallow anything. She immediately received intravenous fluid therapy, antibiotics, and proton pump inhibitors (PPI) and then had her first (and only) dilation on day 22 post-poisoning). The dilation was successful and without complications (such as perforation and bleeding), and the patient was able to swallow liquid drinks. She was discharged without any symptoms. However, 24 h later, she was readmitted to the emergency room with massive oesophageal bleeding, most likely from the arterial fistula. Despite all treatments, she died the same day. We are sure that the patient could have been saved if her parents had not waited for 20 days to seek treatment.

## DISCUSSION

**Figure 1:**
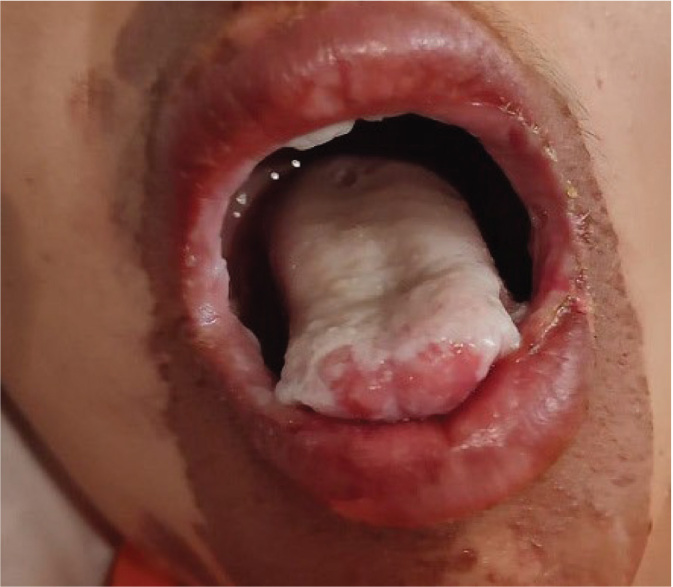
Corrosive damage of a child's tongue and face caused by accidental drinking of white vinegar

To the best of our knowledge, this is the first report ever on white vinegar accidental poisoning in children, who developed oesophageal stricture/stenosis as a consequence. Since the acetic acid concentrations were not known, samples were taken from various households in Hatay and acetic acid concentrations found to reach as high as 98.6 %. Such concentrations can cause severe corrosive damage as shown in Figure 1, including erosion of the oesophageal and gastric mucosa, massive bleeding, gastrointestinal tract perforation, tracheal stenosis, and tracheoesophageal fistula (1, 13, 14, 15). Acetic acid can also cause systemic complications, such as intravascular haemolysis, severe acute kidney and liver failure, and disseminated intravascular coagulation (13). However, these are usually associated with suicidal attempts in adults. What limited evidence we have on accidental children poisoning with acetic acid (9, 14, 16) shows that even a small amount can be lethal due to haemolysis and hepatic dysfunction. However, no systemic effects were observed in our patients. We assume that this is because white vinegar has a very strong odour, which drives children away before they drink too much.

Between the first week and the third week following the ingestion of white vinegar, fibroblast proliferation causes scar and stricture formation in the oesophagus (7, 17). Despite numerous studies of corrosive oesophageal strictures, a standard treatment has not yet been determined (4, 17, 18, 19), but generally it involves replacing food with intravenous fluids, antibiotics, and PPI (4). Partially beneficial effects have been documented with the use of systemic steroids or local steroid or mitomycin C application on the stricture (17). However, there is no consensus among clinicians regarding the initiation time, dose, and method of steroid therapy (7, 17). No local or systemic agent was used in our patients during oesophageal dilation.

Although there are many studies recommending endoscopy early upon hospital admission (24–48 h), there are also those dismissing endoscopy as unnecessary in all cases (1, 12, 13, 18, 20, 21, 22). Those which recommend endoscopy do so because the pH of the substance ingested and the clinical findings often do not correlate with the severity of the burn. Endoscopy can therefore help clinical prognosis, and if there are no burns, the patient can be discharged (1, 12, 13, 20). Those who do not recommend blanket endoscopy refer to symptomatic patients. The severity of the burn will not change treatment, but endoscopy requires general anaesthesia and entails a risk of complications (such as fistulas, perforation, and bleeding) (6, 18, 22). As our patients were all symptomatic (hypersalivation, indications of burns in the mouth), physicians decided that endoscopies were not necessary.

According to several studies (1, 3, 4, 6, 8, 16, 17, 18, 20), the prevalence of oesophageal strictures ranges from 1.72–37 % after ingestion of a caustic substance, and our study is in between with 17.5 %, but our sample is too small for this finding to be considered relevant. Similarly, dilation reported in the above studies was performed 7.15 times per patient in average (from 1 to 40 times) and one study (n=680) reports 82 % success of dilation treatments (15). In our study, dilation was successful in 10 of 11 cases.

## CONCLUSION

We are aware of the small patient sample to draw general conclusions, but these eleven cases still caution us to take accidental ingestion with concentrated white vinegar seriously, especially in countries where acetic acid concentrations in kitchen vinegar are not controlled. The loss of one patient clearly shows that the effects of white vinegar can be deadly in children, especially if parents delay seeking medical help. We also believe that the number of cases can be prevented or at least reduced in developing countries by adopting production control measures such as those used in the European Union or the US and by sealing white vinegar in child-proof containers.
